# Isolation of high-purity muscle stem cells through ice-cold treatment method for the production of cell cultured meat

**DOI:** 10.1016/j.crfs.2025.101253

**Published:** 2025-11-20

**Authors:** Yu-Lin Huang, Zhi-Han Lin, Yan-Qi Song, Zi-Kun Wang, Ling- Ling Weng, Gui-Hai Yang, Nan-Jing Zhong, Guang-Hong Zhou, Yan-Yan Zheng

**Affiliations:** a College of Food Science, Guangdong Pharmaceutical University, Zhongshan, 528458, China; b College of Food Science and Technology, Nanjing Agricultural University, National Center of Meat Quality and Safety Nanjing, MOST, Key Laboratory of Meat Processing and Quality Control, MOE, Key Laboratory of Meat Processing, MOA, Nanjing, 210095, China

**Keywords:** Cell cultured meat, Muscle stem cells, Ice-cold treatment, Large-scale cultivation

## Abstract

Cell cultured meat (CCM) is regarded as a viable alternative in the future meat market due to its traceable origins and environmental friendliness. The production of CCM relies primarily on the efficient *in vitro* expansion of pure muscle stem cells (MuSCs). However, conventional methodologies for isolating MuSCs are often associated with technical complexities, high costs, low cellular purity, and poor stemness of the seed cells. Therefore, this study aimed to use the ice-cold treatment (ICT) method to isolate and purify porcine MuSCs and further characterize their expansion characteristics *in vitro*. The results indicated that cells harvested from unpurified muscle-derived cells treated at around 0 °C for different durations could be cultured *in vitro* for an extended period, while maintaining their differentiation potential. Among them, cells harvested after 30 min of ICT exhibited a high proliferation rate and excellent activity. Furthermore, the MuSCs obtained through this method can be harvested in large quantities through three-dimensional large-scale cultivation in a bioreactor. The harvested cells can subsequently be used to produce meatballs with a texture resembling that of real meat. This method can reduce the cost of obtaining seed cells and accelerate the research on CCM production, thereby providing further inspiration for the industrialized production of CCM.

## Highlights

• Determined the optimal duration for purifying porcine muscle stem cells using the ice-cold treatment method.
• Optimization of the ice-cold treatment method to minimize the loss of primary cells during the purification process, enhancing time efficiency and yield.
• Ice-cold isolated porcine muscle stem cells are stably expanded on microcarriers to produce cultured meat with authentic texture.

## Graphical abstract

**Figure:**
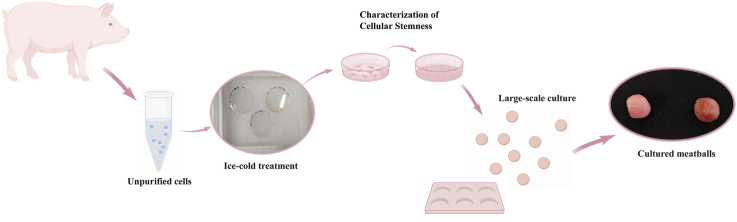

## 1 Introduction

Cell cultured meat (CCM) is regarded as a viable alternative in the future meat market to support the dietary needs of a growing global population. It was one of the top ten breakthrough emerging technologies worldwide in 2018, and has attracted extensive attention due to its traceable origins, green sustainability, as well as animal welfare consideration(Lee et al., 2024; Tomiyama et al., 2020). As early as 1932, British Prime Minister Winston Churchill had predicted that in the future, “it would not be necessary to raise an entire chicken to obtain chicken breast or wings, but rather they could be cultivated through tissue culture”(Ding et al., 2023). In 2013, Mark Post produced the world's first prototype of cultured meat, a beef burger made from bovine MuSCs(Seah et al., 2022). However, its production costs ran into thousands of pounds, involved the assembly of at least 10,000 individual muscle strips(Kadim et al., 2015). Thus, the further expansion of production and commercialization of cultured meat continues to face challenges with regard to both cost as well as technology(Kadim et al., 2015).

MuSCs are regarded as crucial cells for CCM production due to their capacity to precisely form the required muscle tissue with a delicate texture and flavor(Qin et al., 2025). The myotubes differentiated from these cells constitute the primary component of conventional meat(Lee et al., 2023). Maintaining sustained expansion and stable differentiation of MuSCs *in vitro* is crucial for producing CCM(Reiss et al., 2021). Furthermore, previous studies indicate that cell purification plays a vital role in preserving the function of MuSCs(Li et al., 2022; Yoshioka et al., 2020).

The traditional methods for purifying MuSCs primarily comprise three types: the pre-plating method, fluorescence activated cell sorting (FACS) and magnetic activated cell sorting (MACS)(Lee et al., 2024). The pre-plating method is achieved by exploiting differences in cell adhesion properties, as MuSCs exhibit lower adhesion capacity compared to other cells (epithelial cells, fibroblasts). Following enzymatic digestion and dispersion, the cells are inoculated onto uncoated cell culture dishes. By exploiting differences in cell adhesion, MuSCs can be separated from other cells(Sincennes et al., 2017; Wan et al., 2022). Although this method is inexpensive and straightforward to perform, the purity of the cells obtained may fluctuate during culture. Moreover, the stemness of MuSCs is often lost by day 7 due to excessive fibroblast proliferation. FACS is a method for isolating and purifying cells based on specific surface markers possessed by satellite cells(Tierney and Sacco, 2017). Surface markers are proteins highly expressed in specific cells relative to others. Antibodies binding to these proteins are used to label individual cells, which can be subsequently isolated using FACS to obtain pure MuSCs(Basu et al., 2010). The FACS sorting method is the gold standard for cell separation and purification, but it requires substantial quantities of antibodies and a FACS sorter. It is time-consuming, costly, and demands specialized expertise. Concurrently, the process of labelling cells may cause damage to them, which reduces their viability and compromises their stemness. The operating principle of MACS is similar to that of FACS, but MACS employs magnetic fields rather than fluorescent labelling for cell separation(Choi et al., 2025). Its precision is inferior to FACS sorting methods, and the procedure is costly, time-consuming, and causes cellular damage. Furthermore, the purity of the isolated cells may alter during subsequent culture.

MuSCs are considered to possess weaker adhesive capacity than other cell types because of their role as repair cells for skeletal muscle injury. Additionally, they are characterized by strong migratory ability, whereby they respond more sensitively and rapidly to external stimuli(Benedetti et al., 2021; Miyaji et al., 2025; Xu and Velleman, 2023). When a brief ice-cold treatment (ICT; 0 °C, 30min) is applied to cell culture dishes containing heterogeneous monocyte cultures mixed with MuSCs, the MuSCs will float to the top first. This facilitates the separation of MuSCs from other cells, ultimately resulting in the purification of MuSCs. This method involves minimal manipulation of cells, proving more gentle than conventional cell separation and purification techniques, while also causing less damage to the cells. Previous studies have demonstrated that low temperatures reduce the expression of cell surface adhesion molecules, thereby leading to cell detachment(Juliano and Gagalang, 1979; Rico et al., 2010). Moreover, as early as 2008, Beaty and colleagues proposed the possibility of using cold treatment to separate cells(Kozanoglu et al., 2008). Thereafter, Benedetti et al. successfully isolated pure mouse and human MuSCs using the ICT method(Benedetti et al., 2021), although they did not investigate isolation conditions for MuSCs from other species.

Therefore, the present study adopted an ICT method to isolate and purify porcine MuSCs and investigate the functional characteristics of cells harvested after undergoing varying durations of ICT. Subsequently, the harvested porcine MuSCs were scaled up using a bioreactor for culture, ultimately yielding a cultured meat product with a texture similar to conventional meat. Overall, this study aimed to provide theoretical support for improved industrial production of CCM.

## 2 Materials and methods

### 2.1 Porcine primary cells isolation

Isolation of primary porcine cells predominantly involved harvesting the biceps femoris and erector spinae muscles from one-week-old piglets. These were immersed in a 70 % (v/v) ethanol solution for 1–2 min. Thereafter, the meat was then minced under sterile conditions to dimensions of 0.5–1.5 mm^3^ and the minced tissue was placed in DMEM/F12 medium (Gibco, China) supplemented with 1 % penicillin/streptomycin (v/v) (Gibco, USA) at 37 °C. Additionally, collagenase (Sigma, USA) and dissipative enzyme (Roche, Switzerland) were used to digest the muscle tissue. During digestion, the sample was gently blown through the syringe using a syringe plunger. The endpoint of digestion was determined when the digested cellular material could be readily passed through the syringe needle. Following digestion, the cells were centrifuged at 900 g for 5 min and subsequently isolated. Thereafter, the digestion mixture was added through a 100 μm cell strainer. Red blood cell lysis buffer was added to the cells in the pellet and they were incubated on ice for 5 min to lyse the red blood cells. Finally, the solution was filtered through a 40 μm cell strainer and centrifuged again (900 g, 5 min) to collect the cells intended for purification.

### 2.2 Porcine MuSCs purification

Primary unpurified porcine cells were seeded onto 100 mm uncoated cell culture dishes. Based on prior research by Li and Wang(Li et al., 2022), pre-plating for 0.5 h yielded optimal cell purification results, so the present study opted for the same pre-plating period. The non-adherent cells were initially collected and resuspend in DMEM/F12 containing 15 % fetal bovine serum (FBS) (Gibco, USA). Subsequently, they were seeded onto a 100 mm cell culture dish (Jet, China) covered with 0.05 % mouse tail collagen (Corning, USA) and incubate at 37 °C in 5 % CO_2_ for 20 h. The following day, the medium was discarded and the adherent cells were washed twice in the culture dish with 8 mL of phosphate-buffered saline (PBS) (Biosharp, China). Subsequently, 4 mL of DMEM/F12 (pre-chilled to 4 °C) was added to the cells. The culture dish was then placed in an ice bath at 0 °C for 20 min, 30 min, and 40 min, accompanied by occasionally gentle shaking to aid cell detachment. Thereafter, the samples were centrifuged at 300 g for 5 min to collect the exfoliated cells, which were the purified porcine MuSCs.

### 2.3 MuSCs culture and differentiation

The proliferation medium for MuSCs consisted of DMEM/F12 supplemented with 15 % FBS, 1 % (v/v) penicillin/streptomycin, and 5 ng/mL basic fibroblast growth factor (GenScript, China). Similarly, the differentiation medium comprised DMEM/F12 supplemented with 2 % horse serum and 1 % (v/v) penicillin/streptomycin. The purified porcine MuSCs were cultured in cell culture dishes covered with mouse tail collagen, supplemented with proliferation medium to provide the nutrients required for cell growth.

To differentiate the porcine MuSCs, the purified cells were seeded into culture dishes coated with 1 % Matrigel (Corning, USA). Upon reaching over 100 % confluence, the medium was replaced with differentiation medium to induce cellular differentiation. Expanded and differentiated cells were lysed using RIPA buffer (Beyotime, China) supplemented with PMSF (Beyotime, China) to collect proteins for Western blot analysis.

### 2.4 Cell viability

MuSCs at P3 with different ICT durations were seeded at 5 × 10^3^ cells per well into 96 wells of cell culture dishes covered with 0.05 % mouse tail collagen and cultured at 37 °C. After 24 h, 10 μL of CCK8 reagent was add to the cell culture medium, followed by incubation at 37 °C for 2 h. Thereafter, the optical density (OD) values of each group were measured at 450 nm using a microplate reader (BioTek, USA).

### 2.5 SA-β-gal activity

The SA-β-gal activity in MuSCs obtained from different ICT durations at the P5 was analyzed using the SA-β-gal staining kit (Vazyme, China) according to the manufacturer's instructions. After culturing cells in a 12-well plate covered with mouse tail collagen for 1 day, the medium was discarded. Subsequently, the cells were washed with PBS, followed by the addition of fixative solution and fixation at room temperature (25 °C) for 15 min. SA-β-gal staining solution was then added to the cells and they were incubated overnight at 37 °C in a CO_2_-free environment, protected from light. Subsequently, images were acquired directly using the Olympus CKX41 fluorescence inverted microscope.

### 2.6 Immunofluorescence analysis

The cells were washed twice with PBS, then fixed them with 4 % Paraformaldehyde (PFA) (Beyotime, China) at room temperature for 20 min or overnight at 4 °C. After washing away the fixative with PBS, the cells were permeabilized at room temperature with 0.5 % Triton X-100 for 20 min. Subsequently, they were blocked with 1 % Bovine Serum Albumin (BSA) at room temperature for 1 h, followed by the addition of diluted primary antibody mouse myosin heavy chain (*MyHC*) (1:300, Abcam, ab37484) and overnight incubation at 4 °C. Thereafter, the cells were washed thrice with PBS and the secondary antibody (1:100, Affinity, USA) was added. This was followed by incubation at room temperature for 1 h. The cells subsequently underwent nuclear staining for 8 min using DAPI stain (Servicebio, China) at a final concentration of 2 μg/mL. Finally, the samples were covered using anti-fade mounting medium (Servicebio, China) and imaged using the Olympus CKX41 fluorescence inverted microscope.

### 2.7 Gene expression analysis

RNA was extracted and purified from the cells using a total RNA extraction kit (Vazyme, China). Thereafter, the RNA concentration was determined using a microvolume spectrophotometer (Denvir DS-11, UK), followed by reverse transcription of the RNA into cDNA using the Prime Script RT Master Mix (Takara Bio, Japan) according to the manufacturer's instructions. Quantitative RT-PCR (qRT-PCR) was performed in triplicate using the ChamQ SYBR qPCR Master Mix (Vazyme, China) in a real-time fluorescent quantitative PCR instrument (Bio-Rad CFX96 Touch, USA), with *GAPDH* serving as the housekeeping gene and normalization to the control level using the 2^−ΔΔ Ct^ method. The primers used in these assays were as follows:

*PAX7* -F: GTGCCCTCAGTGAGTTCGATT.

*PAX7* -R: TCCAGACGGTTCCCTTTGTC.

*MYOG*-F: AACCCCACTTCTATGACGGG.

*MYOG*-R: TTATCTTCCAGGGGCACTCG.

*MYOD* -F: GCTCCGCGACGTAGATTTGA.

*MYOD* -R: GGAGTCGAAACACGGGTCAT.

*MyHC*-F: AGGACCAAGTACGAGACGGA.

*MyHC*-R: AGCTTCCACGTGTTCCTCAG.

*GAPDH*-F: TGAGATCCAGGGAGCCATCA.

*GAPDH*-R: ATGGTCAGGGGTCCGAT-GTA.

### 2.8 Western blot analysis

Cells were lysed using RIPA buffer supplemented with 1 % PMSF to extract proteins, followed by the determination of protein concentration was determined using the BCA protein assay kit (Thermo Fisher, USA). The proteins were loaded into the gel wells and transferred onto a PVDF membrane via electrophoresis. Subsequently, the membrane was blocked with TBST (Servicebio, China) containing 5 % skim milk and incubated at 4 °C for 12 h with appropriately diluted primary antibodies against *PAX7* (1:100, DSHB, Cat#AB_528428), *MyHC* (1:2000), and *GAPDH* (1:1000, Biosharp, Cat#BL072A). Following this, the secondary antibody (HRP-conjugated goat anti-mouse IgG, Biosharp, Cat#BL001) was incubated at room temperature for 2 h. The chemiluminescent HRP substrate and the Chemiluminescence imaging system SCG-W3000(Servicebio, China) were used for imaging.

### 2.9 Expansion culture of MuSCs

Approximately 5 g of microcarriers (MCs) (Cytodex, USA) were dispersed in 3.5 mL of proliferation medium, and the mixture was subsequently transferred to a sterile ultra-low-adhesion six-well plate. Thereafter, the purified porcine MuSCs were seeded at a density of 8 × 10^3^ cells/cm^3^ onto the MCs. The MuSCs were gently mixed with the MCs by pipetting to facilitate better cell adhesion to the MCs. The cell mixture was cultivated for 7 days and harvested once the MuSCs and MCs had expanded.

### 2.10 Live/dead cell staining

First, 0.5 g of MCs were collected from the ultra-low adsorption six-well plate and the supernatant was discarded to isolate the MCs with adhered cells. Following the manufacturer's instructions, the cells were incubated in the dark for 30 min after using the Cytotoxicity Assay Kit (Beyotime, China) for live-dead cell staining.

### 2.11 Cooking loss

The cells cultured on the MCs were harvested along with the carriers and mixed with starch to produce meatballs. These were steamed for 3 min at 100 °C, followed by cooling to 25 °C. Cooking loss was expressed as a percentage of sample weight and calculated according to the following formula:

$$\text{Cooking}\;\text{Loss}\left(\%\right)=\frac{\text{Raw}\;\text{Weight}\left(g\right)\;‐\;\text{Cooked}\;\text{Weight}\left(g\right)}{\text{Raw}\;\text{Weight}\left(g\right)}$$

### 2.12 Textural properties

Textural profile analysis (TPA) was performed on the produced meatballs using the TA. XTplus software (TA.XTplus, Stable Micro Systems, UK). First, the textural properties of the cultured meatballs and real meatballs were measured using a 5 mm cylindrical probe. The experimental parameters were set as follows: pre-test speed:1.0 mm/s, test speed:1.0 mm/s, post-test speed: 1.0 mm/s, strain:50 %.

### 2.13 Statistical analysis

The data have been expressed as the mean ± standard deviation (M ± SD). The statistical analysis was performed using GraphPad Prism 10. The *t*-test was used to compare two experimental groups, whereas one-way ANOVA with Tukey's multiple comparison test was applied to three or more experimental groups. The differences among indicators were indicated using letter-based labeling and the statistical significance level was defined as P < 0.05.

## 3 Results and discussion

### 3.1 Isolation of cells using the ICT method and characterization of their viability

**Fig. 1:**
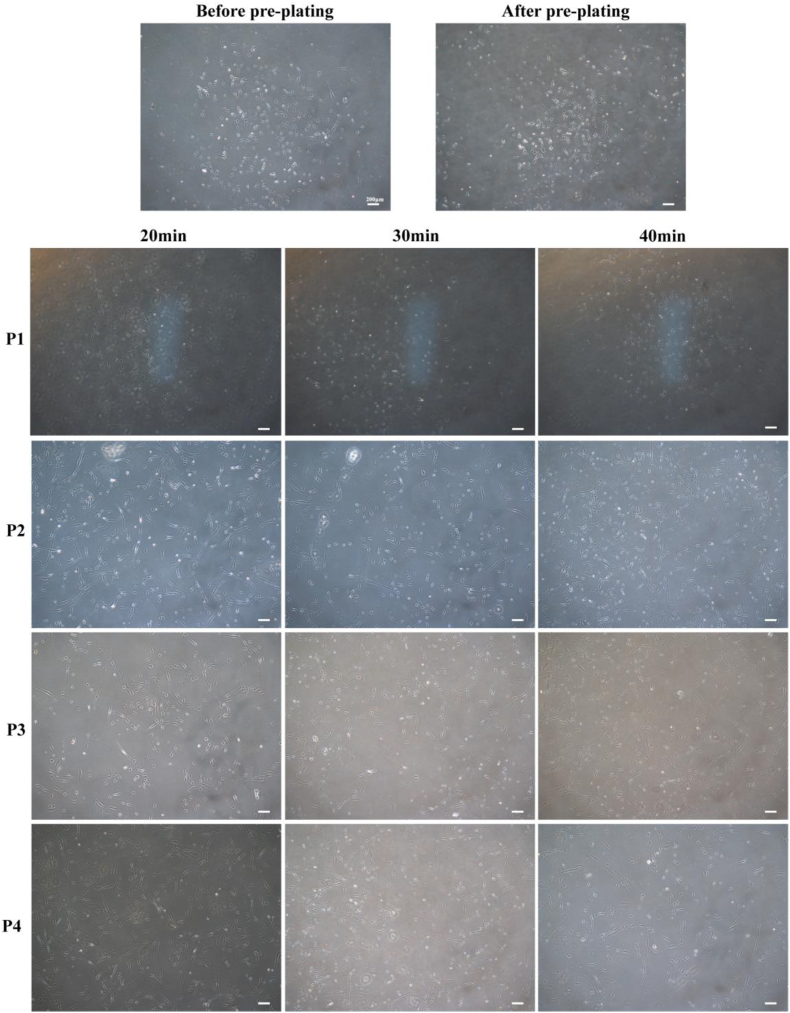
Brightfield micrographs of cells harvested after varying durations of ICT method Note: Brightfield images of cells cultured from P1 to P4 passages, obtained after 20min,30min and 40min ICT method. The scale bar was 200 μm.

**Fig. 2:**
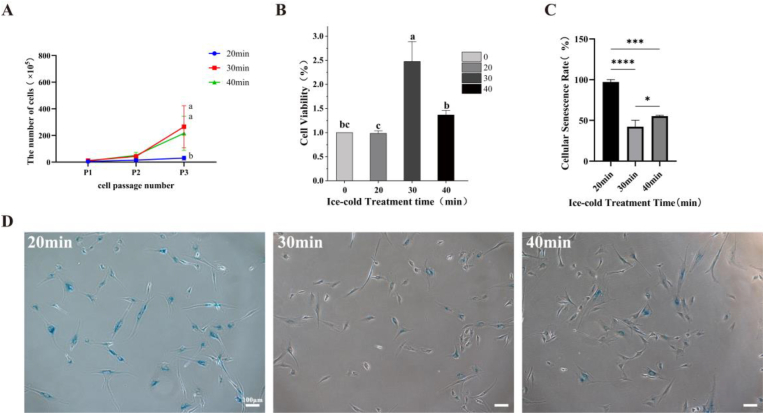
The activity of cells and the cell harvest quantity after varying durations of ICT method Note:(A) Proliferation curve of cells cultured from P1 to P3 passages (n = 3). (B) Cell viability of purified MuSCs obtained at different ICT duration, with MuSCs isolated by FACS serving as the control group (n = 4). (C–D) Figure C shows the quantitative results of SA-β-gal staining. Figure D shows a representative image of SA-β-gal staining (n = 3). The scale bar was 100 μm.

Previous research has demonstrated that the purification and cultivation of MuSCs holds significant importance for the study of MuSCs and their subsequent applications(Frimand et al., 2022; Yoshioka et al., 2020). This is due to the superior proliferative capacity of other cells (fibroblasts), which leads to extensive fibroblasts contamination of MuSCs cultures during later stages of cultivation and compromises their differentiation potential. CCM primarily relies on the *in vitro* expansion and differentiation of MuSCs to produce more animal proteins during meat production(Danoviz and Yablonka-Reuveni, 2012; Gharaibeh et al., 2008; Li et al., 2022). Cell adhesion relies on the support of adhesion sites; low temperatures reduce extracellular matrix secretion, and cause diminished cell adhesiveness(Hirasawa et al., 1994; Jetha et al., 2007; Marlin and Springer, 1987). MuSCs are the first resident cells to respond to injury, exhibiting strong migratory capacity and sensitivity to stimuli, and are thus considered to possess low adhesiveness(Duran et al., 2024; Hüttner et al., 2021; Wang and Rudnicki, 2012). Benedetti et al. experimentally provided preliminary confirmation of this supposition(Benedetti et al., 2021). To determine the optimal time for purification of porcine primary cells via the ICT method, the pre-adherent pig primary cells were initially placed on ice to stimulate the primary cell suspension. These were subjected to ICT for 20, 30, and 40 min respectively, after which the floating cells were collected to yield purified porcine MuSCs. Subsequently, the MuSCs were seeded in cell culture dishes and cultured to the P4 stage. As observed in the brightfield images (Fig. 1), all three cell groups exhibited favorable cellular conditions and rapid division rates during the initial culture phase. Furthermore, the proliferation of MuSCs were quantified from passage P1 to P3. During the culture period, cells obtained after 30 min of ICT exhibited significantly higher growth multiples than the other two groups. In contrast, cells from the 20 min ICT group demonstrated the lowest proliferation rate among the three groups (Fig. 2-A). The brightfield image also revealed that when the MuSCs obtained from the ICT were further cultivated to the later stage, the MuSCs obtained after 30 min of the ICT still maintained good viability when cultured to the P4 stage. Moreover, in late-stage cultured cells, the nuclear-to-cytoplasmic ratio of cells obtained after 30 min of ICT was higher than that of cells obtained after 20 or 40 min of treatment. Previous studies indicate that a higher nuclear-cytoplasmic ratio correlates with higher cellular metabolic activity, which is indicative of better cell vitality(Biswas et al., 2025; Colgren and Burkhardt, 2023). This suggests that the 30 min group exhibited superior cellular viability and demonstrated the strongest proliferative capacity among the three cell groups. Concurrently, cells subjected to a 20 min ICT exhibited the poorest performance across all three cell types. CCK8 assays performed on P3 cells further revealed (Fig. 2-B) that the MuSCs obtained via 20 min ICT demonstrated significantly lower viability compared to the MuSCs from the other two ICT groups. Under brightfield microscopy, the cells exhibited the lowest nucleus-to-cytoplasm ratio, along with elongated, flattened forms, and slow growth. This may be attributed to the limited number of cells obtained after a mere 20 min ICT, coupled with premature senescence resulting from the cells having undergone multiple divisions by the time they reached the P4 stage after three generations(Blau et al., 2015). Compared to MuSCs isolated via FACS, cells obtained after 30 min of ICT exhibited significantly higher viability (*P* < 0.05). This difference relates to the methods employed for cell purification. FACS requires prior labelling of cell surface receptors, whereas the ICT method appears gentler, involves fewer manipulations, and causes less stress to the cells during purification, thereby yielding higher viability(Jager et al., 2018).

To further verify whether the differences in the proliferative capacity of MuSCs during the later stages of culture were associated with cellular senescence, this study also detected senescence in P5 cells obtained after being subjected to different ICT durations via senescence-associated β-galactosidase (SA-β-gal) staining (Fig. 2C–D). It was observed that the proportion of cells stained in the 20 min ICT group was significantly higher than that of the other two groups, whereas the staining rate was lowest for cells obtained from the 30 min ICT group (*P* < 0.05). This indicates that cells obtained after undergoing 20 min of the ICT method exhibited widespread senescence during later stages of culture, which led to reduced cellular activity. Conversely, cells obtained after 30 min of the ICT method demonstrated superior maintenance of cellular viability throughout the culture process. Overall, this finding was consistent with the results of the preceding experiments.

### 3.2 Differentiation capacity of MuSCs purified by the ICT method

**Fig. 3:**
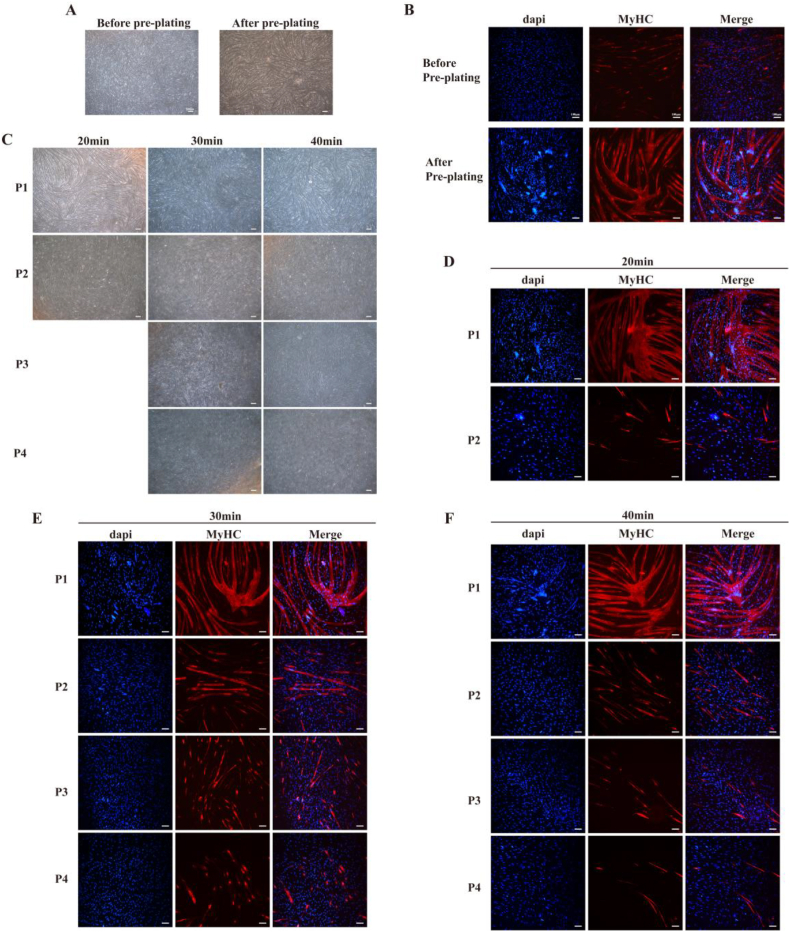
The differentiation of muscle tubes in cells after varying durations of ICT Note:(A) Brightfield images of myotube differentiation before and after pre-plating. (B) Pre-plating cells immunofluorescence staining images. (C) Brightfield images of cell differentiation from P1 to P4 generations obtained at different ICT method durations. (D–F) Immunofluorescence images of cell differentiation from P1 to P4 generations. Blue represents DAPI, red represents MyHC. The scale bar for brightfield images of cell differentiation is 200 μm, while that for immunofluorescence staining images is 100 μm.

To investigate the differentiation potential of purified MuSCs, cellular differentiation was induced, during which the cells progressively elongated and fused to form myotubes. After pre-plating, the myotube area in brightfield microscopy was markedly greater than that observed prior to pre-plating. Immunofluorescence results also demonstrated a higher *MyHC* expression rate in MuSCs after pre-plating, along with a higher fusion rate and the formation of more robust myotubes (Fig. 3A–B). Both brightfield observations of cell differentiation as well as immunofluorescence staining results confirmed that pre-plating effectively achieved preliminary cell purification.

Furthermore, cells obtained via the ICT method were cultured and induced to differentiate. Observation under brightfield microscopy (Fig. 3 C) revealed that these cells could effectively differentiate at the early stage of cultivation. After only 3 days of induction differentiation, the formation of several thick myotubes could be observed. Unfortunately, cells obtained after 20 min of ICT could no longer be expanded to a density suitable for differentiation beyond the P3 stage, thereby indicating loss of their differentiation capacity. Moreover, the differentiation efficiency at P2 was significantly reduced compared to the first passage. With increasing passage numbers, all cell groups exhibited a decline in differentiation capacity. This may be attributed to the absence of the physicochemical niche associated with MuSCs in the *in vitro* culture environment, which impairs intracellular signaling pathways. This finding was also consistent with previous studies.

Notably, the immunofluorescence staining experiments revealed (Fig. 3D–F) that the differentiation capacity of cells obtained from the three ICT groups exhibited variations with increasing passage numbers. Cells obtained after undergoing 30 min of ICT maintained a favorable differentiation capacity during serial passage. Furthermore, MuSCs from the 30 min ICT group demonstrated superior preservation of their differentiation potential compared to the other two groups. This may be attributed to the insufficient number of MuSCs obtainable after only 20 min of ICT. Consequently, by P3, these cells had undergone excessive divisions, leading to loss of stemness and subsequent failure in cell fusion, which prevented further myotube formation. However, due to the prolonged cooling duration in cells obtained after 40 min of ICT, they were not only enriched for MuSCs but also collected alongside other cell types such as fibroblasts. During later stages of culture, fibroblasts proliferated rapidly, whereby they overgrew and contaminated the MuSCs. As a result, cells harvested after 40 min of ICT can only differentiate into tiny myotubes and exhibit significantly lower differentiation efficiency compared to the cells obtained after undergoing 30 min of ICT. Chen et al. investigated the transcriptional profiles of porcine cells during early and late stages of culture(Chen et al., 2025). They found that the proportion of fibroblasts increased in porcine skeletal muscle cell cultures during the later phase, indicating that these cultures are often contaminated by fibroblasts during the culture process. Similarly, cells obtained after 40 min of ICT, contained a higher initial number of fibroblasts during culture and exhibited excessive proliferation that led to the contamination of MuSCs. This contamination persisted into the later stages of the culture process, which resulted in the cells obtained after 40 min of ICT to only differentiate into tiny myotubes. Moreover, the differentiation efficiency was significantly slower than that of the cells treated for only 30 min.

### 3.3 Gene and protein expression in MuSCs obtained via the ICT method

**Fig. 4:**
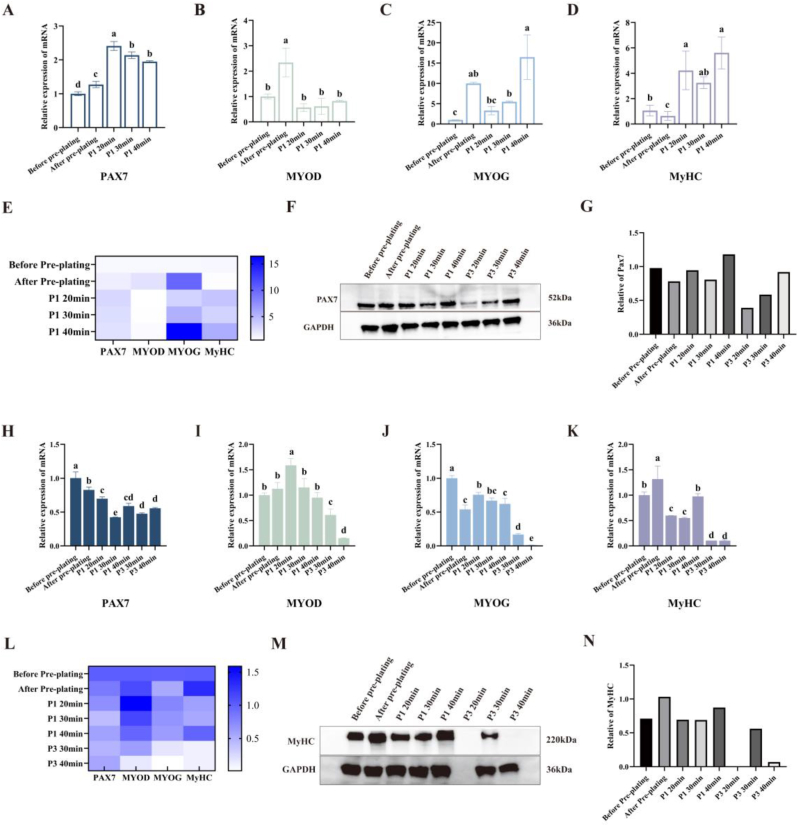
Expression profiles of cellular genes and proteins following varying duration of ICT Note:(A–D) The relative mRNA expression levels of PAX7, MYOD, MYOG, and MyHC in proliferating-stage cells were analyzed by qRT-PCR (n = 5). (E) Heatmap of gene expression levels during cell proliferation stages obtained via pre-plating and ICT method. (F–G) Western blot analysis revealed the protein expression levels of PAX7 and GAPDH in cells during the stage of proliferation, along with statistical data on relative protein expression. (H–K) Relative mRNA expression levels of PAX7, MYOD, MYOG, and MyHC in differentiated-stage cells (n = 5). (L) Heatmap of gene expression levels in differentiated-stage cells obtained via pre-plating and ICT method. (M) Expression levels of MyHC and GAPDH proteins during cellular differentiation, along with statistical data on relative protein expression.

Based on our previous investigation into the proliferation and differentiation capacity of MuSCs, a 30 min duration appeared to be the optimal time for isolating pure MuSCs through the ICT method. Therefore, this study further examined the gene expression profiles of the cells purified by the ICT method for three different durations. Following ICT purification (Fig. 4A–E), all cells exhibited elevated *PAX7* expression (*P* < 0.05), with MuSCs obtained after the 20 min ICT demonstrating the highest expression levels and consequently indicating the highest degree of purification. Among the five cell groups, cells before and after pre-plating exhibited increased *MYOD* expression, whereas cells subjected to ICT demonstrated increased expression of *MYOG* and *MyHC* (*P* < 0.05). Therefore, cells purified by the ICT method appeared to exhibit superior stemness. During the early stages of cultivation, not only can stem cell genes such as *PAX7* be highly expressed, but also other genes such as *MYOG* and *MyHC*(Mohan et al., 2023). This is because cells obtained after ICT exhibit high purity and high stemness as MuSCs, thereby enabling the robust expression of animal myogenic genes such as *MYOG* and *MyHC*. Furthermore, Western blot results (Fig. 4F–G) indicated that ICT cells exhibited higher *PAX7* expression levels than the after pre-plating cells, which was also consistent with the qRT-PCR findings. In P3 cells, the protein expression level of *PAX7* exhibited a downward trend. Among them, the cells subjected to ICT for 20 min demonstrated the greatest decline, which was related to the earlier occurrence of senescence in these cells. Among the three groups of cells subjected to ICT, the *PAX7* protein expression levels in MuSCs treated for 20 and 30 min were lower than those in cells treated for 40 min. This discrepancy arose from the temporal lag between mRNA and protein expression(Reimegård et al., 2021). Cells obtained after undergoing 40 min of ICT had progressed to the protein accumulation phase for *PAX7* expression, whereas MuSCs from the 20 and 30 min ICT groups predominantly retained *PAX7* expression at the mRNA stage. This temporal difference constituted a key factor in explaining the disparity in gene and protein expression observed between the 30 and 40 min ICT groups.

Product manufacturing often requires the use of cells that have been cultivated to a later stage(Pasitka et al., 2023). Consequently, the present study incorporated assessments of gene and protein expression in cells at the P3 differentiation stage. However, cells obtained following 20 min of ICT cannot undergo P3 differentiation stage analysis due to premature senescence. To assess the differentiation ability of these cells, genetic testing was performed on the MuSCs differentiated at P1 and P3 (Fig. 4H–L). The expression level of *MYOG* in the P1 generation cells that were subjected to ICT for 40 min was lower than those that underwent ICT for 20 min yet demonstrated higher *MyHC* expression than cells subjected to 20 min or 30 min of ICT (*P* < 0.05). This indicates that the cells obtained after ICT for 40 min in the P1 generation were more mature in differentiation and had higher expression levels of genes related to myogenesis. Concurrently, as the cells purified via the ICT method predominantly comprise MuSCs, their stemness genes may remain highly expressed even after three consecutive passages of expansion. As a result, following induced differentiation, the expression levels of the *PAX7* gene do not exhibit a significant decline.

In both groups of P3 cells, there was no significant difference in *MyHC* expression levels. However, MuSCs obtained after 30 min of ICT exhibited higher *MYOD* and *MYOG* expression levels compared to those treated for 40 min (*P* < 0.05), thus indicating that MuSCs from the 30 min ICT group demonstrated superior differentiation potential. Furthermore, Western blot analysis (Fig. 4M–N) revealed that cells from the 30 min ICT group maintained superior differentiation capacity even during later stages of culture. Consequently, MuSCs obtained from cells subjected to the 30 min ICT demonstrated superior differentiation maintenance compared to those from the 40 min ICT group, which was consistent with the qRT-PCR findings.

However, following ICT, cells exhibited upregulation of *PAX7* gene expression, yet after induced differentiation, their *MyHC* expression levels were lower than those of cells merely after pre-plating. This indicates that cryopreservation causes damage to stem cells that cannot be overlooked(Griessinger et al., 2023; Yildiz et al., 2021). Previous studies have demonstrated that cold exposure can cause dysfunction in Schwann cell nerve signaling, consequently impairing cellular function and triggering disease(Bai et al., 2023). Although cryopreservation is a gentler method compared to techniques such as FACS or MACS, it may still lead to mitochondrial damage, reactive oxygen species production, and the accumulation of lipid peroxides during the cold treatment process. These are considered the primary cytotoxic effects of cold stress, which impair the myogenic potential of the cells(Kozanoglu et al., 2008; Li et al., 2024; Vairetti et al., 2001). Consequently, cells obtained after ICT following induced differentiation exhibit inferior myogenic capacity compared to those observed merely after pre-plating.

Previous studies have demonstrated that longer periods of ICT yield greater numbers of initially obtained stem cells, yet the purity of the acquired MuSCs diminishes. Compared with the other two groups, the cell purity obtained after 20min of ICT was higher. Therefore, the expression level of the *PAX7* gene specifically expressed by MuSCs was also elevated. MuSCs obtained after 40 min of ICT exhibited greater initial cell numbers, facilitating differentiation. Consequently, following initial differentiation induction, *MyHC* expression levels were higher in these MuSCs compared to those from 20 min or 30 min ICT groups. However, as the culture progressed, the excessive proliferation of contaminating cells obtained after the 40 min ICT contaminated the MuSCs. The differentiation capacity of the ICT cells thus declined significantly following the induction of P3 cells. Cell cultures isolated by FACS also exhibited a tendency for earlier-passage cells to differentiate more readily than later-passage cells, further demonstrating that MuSCs become contaminated by other cell types or lose their stemness during prolonged culture(Choi et al., 2025; Jager et al., 2018). This confirms that a 30 min ICT alone effectively balances the numbers of contaminating cells and MuSCs, thereby proving most advantageous for maintaining MuSC stemness.

### 3.4 Expansion of MuSCs on microcarriers

**Fig. 5:**
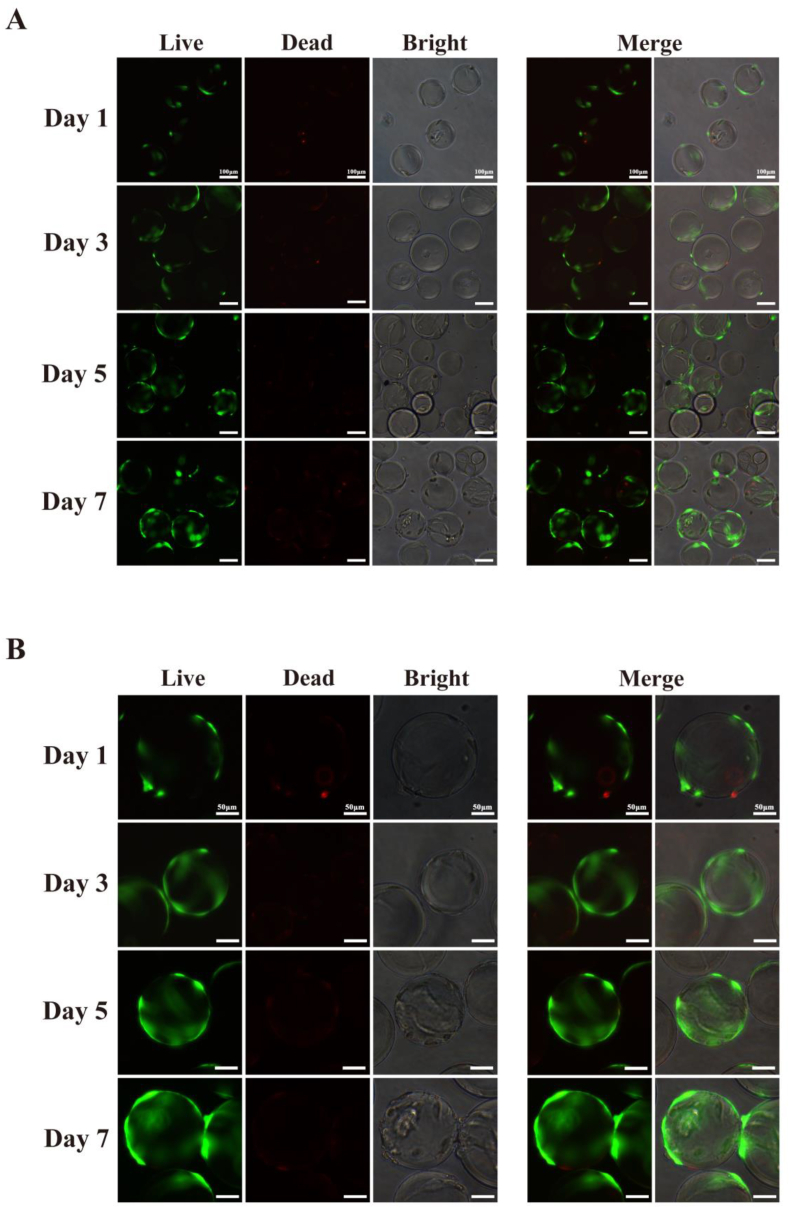
The purified MuSCs were expanded and cultured on microcarriers. Note: (A) Representative live/dead cell staining at 100 × magnification. The scale is 100 μm. (B) Representative live/dead cell staining at 200 × magnification. The scale is 50 μm. Green indicates live cells (calcein, AM); red indicates dead cells (propidium iodide, PI).

For the production of cultured meat, cells often need to be expanded in three dimensions to sufficient numbers (Kaneko et al., 2023; Pasitka et al., 2023). Therefore, this study we further investigated whether MuSCs obtained via the ICT method could effectively adhere to MCs and proliferate normally. MuSCs obtained after the 30 min ICT were seeded onto commercial MCs and live/dead staining was performed every 2 days (Fig. 5A–B). Staining results demonstrated that over the 7 days of culture, the green area in the live/dead staining images progressively increased, thus indicating a rise in viable cell numbers. Furthermore, by day 7, adhesion occurred between MCs due to the high cell density on the MCs. This may be attributed to the extracellular matrix secreted by MuSCs(Gao et al., 2023; Rmaidi et al., 2021). Collectively, this indicates that the cells adhered and proliferated effectively on the MCs, and the cells obtained via the ICT method were suitable for CCM production.

### 3.5 Characteristics analysis of CCM

**Fig. 6:**
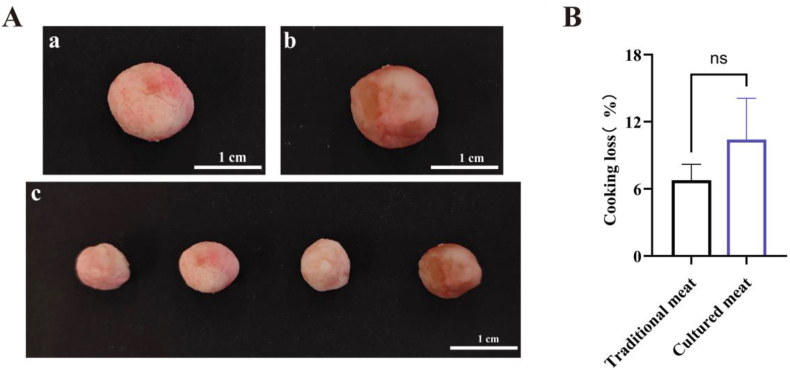
Image of CCM products produced from MuSCs obtained through ICT method Note:(A) Brightfield images of cultured meatballs made from cells harvest after microcarrier expansion culture, as well as images of real meatballs. The scale bar was 1 cm. The letter a shows cultured meatball, b shows real meatball, and c is a brightfield image of cultured meatballs and real meatballs photographed together. (B) Cooking loss of cultured meat and traditional meat (n = 5).

Muscle tissue provides ample amino acids and chewiness in meat(Ellulu et al., 2015). Mainstream consumers have demonstrated increased willingness to purchase cultivated meat that contains muscle tissue(Rubio et al., 2020). Therefore, the present study blended MCs containing MuSCs with starch to create cultured meatballs by mimicking traditional meatball production methods (Fig. 6 A). Subsequently, the physical properties of this cultured meat was examined. Textural analysis revealed (Table 1) that cultured meatballs were indistinguishable from those made from conventional pork with regard to their hardness (*P* < 0.05). However, owing to the former relying solely on starch for structural support and exhibiting lower crosslinking, their stickiness and chewiness were inferior to those of traditional pork meatballs. Furthermore, no significant difference was observed in the loss incurred by both types of meatballs during steaming (Fig. 6 B). Overall, cultured meatballs were found to be comparable to conventional pork meatballs across various properties.

**Table 1:** Textural properties of raw traditional meatballs and cultured meatballs.

	Traditional meatballs	Cultured meatballs
Hardness(N)	95.287 ± 45.994^a^	129.766 ± 38.019^a^
Springiness	0.573 ± 0.192^a^	0.633 ± 0.181^a^
Cohesiveness	0.501 ± 0.091^a^	0.256 ± 0.048^b^
Gumminess	45.035 ± 17.272^a^	32.840 ± 10.822^b^
Chewiness(N)	27.164 ± 16.561^a^	20.936 ± 10.052^b^
Resilience(N)	0.283 ± 0.065^a^	0.203 ± 0.031^a^

Note: A total of six indicators within the texture properties were analyzed in this analysis. Results are presented as the mean ± SD, n = 5.

## 4 Conclusion

This study investigated the optimal timing for isolating porcine MuSCs using the ICT method. MuSCs obtained after 30 min of cold treatment demonstrated the greatest potential for maintaining their myogenic capacity during long-term culture. Compared to isolating MuSCs via FACS or pre-plating methods, the ICT approach proved less time-consuming, more convenient, and yielded cells capable of expansion on MCs. Although the ICT method appears gentler than traditional cell purification techniques such as FACS, the cellular damage caused by cold stimulation remains significant. Overall, this study provided theoretical support for efficiently obtaining seed cells for CCM, thereby reducing production costs. It contributed to the low-cost, industrial-scale production of CCM.

## CRediT authorship contribution statement

**Yu-Lin Huang**: writing–review and editing, writing–original draft. **Zhi-Han Lin**, **Yan-Qi Song**: writing–original draft. **Zi-Kun Wang**, **Ling-Ling Weng**, **Gui-Hai Yang**: review and editing. **Yan-Yan Zheng**: writing–review and editing, writing–original draft. **Nan-Jing Zhong**: writing–review and editing, writing–original draft. **Guang-Hong Zhou**: review and editing, writing–original draft.

## Declaration of competing interest

The authors declare that there are no conflicts of interest.
